# Acute Surgical Abdomen: An Unusual Presentation of Pulmonary Embolus

**DOI:** 10.1177/2324709614542339

**Published:** 2014-07-27

**Authors:** Nader AL-Mane, Faisal AL-Mane, Zein Abdalla, Joe McDonnell

**Affiliations:** 1 Naas General Hospital, Naas, Co. Kildare, Ireland; 2 Woodstock General Hospital, Woodstock, Ontario, Canada

**Keywords:** massive pulmonary embolism, surgical abdomen, cardiac arrest, abdominal pain, laparotomy

## Abstract

*Background*. Pulmonary embolism is a common and potentially lethal condition. Most patients who die from massive pulmonary embolism do so within the first few hours of the event. The clinical manifestations of pulmonary embolism are nonspecific, which makes the diagnosis difficult. *Case Report*. We present a case of massive pulmonary embolism presenting as an acute surgical abdomen that underwent exploratory laparotomy and made a complete recovery. *Why should an emergency physician be aware of this?* Emergency department physicians should be aware that massive pulmonary embolism could present as an acute surgical abdomen in young healthy individuals.

## Introduction

Massive pulmonary embolism (PE) is a potentially fatal condition and many patients die within the first few hours of its occurrence. Clinical manifestations are nonspecific, which makes the diagnosis difficult. Acute surgical abdomen and distension is not commonly listed as presenting features of PE.

## Case Report

A 35-year-old healthy, nonsmoker female school teacher previously on an oral contraceptive pill (co-cyprindiol) for the last 2 years used for severe acne presented to the emergency department of a district hospital by ambulance after collapsing at her workplace with severe epigastric pain, periumbilical pain, and gross abdominal distension of 1 hour duration. She denied dyspnea or chest pain. On examination she was in extreme pain and bending her knees. Her vital signs on admission were as follows: blood pressure 154/67, pulse rate 123 per minute sinus rhythm, O_2_Sat 98% on room air, respiratory rate 27 per minute, and temperature of 34.3°C. A physical examination of the chest was unremarkable, but abdominal exam revealed a tender distended abdomen with hepatomegaly. Pantoprazole 40 mg and morphine 5 mg were administered intravenously.

Urine pregnancy test was not possible because patient was anuric, so a serum HCG was sent to a tertiary center. Electrocardiogram showed sinus tachycardia 123 per minute with no evidence of S1Q3T3. Abdominal ultrasound 1 hour after admission showed free fluid in the upper abdomen and around the inferior border of both liver and spleen. As the bladder was empty, it was not possible to do an ultrasound of pelvis. Routine laboratory testing on arrival in the emergency department revealed the following results: hemoglobin (Hb) 11.7 g/dL, white cell count 11 × 10^9^/L, platelets 125 × 10^9^/L, coagulation profile prothrombin time (PT) 13 (international normalization ratio [INR] 1.1), activated partial thromboplastin time (APTT) 35 (APTT ratio 1.2), creatinine 131 µmol/L, urea 4.3 mmol/L, and potassium 3.1 mmol/L. Liver function tests: albumin 34 g/L, alanine aminotransaminase (ALT) 28 U/L, alkaline phosphatase 41 U/L, and amylase 36 IU/L. pH 7.12, PaCO_2_ 4.59 kPa, PaO_2_ 8.32 kPa on 1.0 FiO_2_, HCO_3_ 12.9 mmol/L, BE negative 15.5, and normal troponin. The patient developed asystolic cardiac arrest 90 minutes after presentation to the emergency department and received 2 minutes of cardiopulmonary resuscitation with chest compressions, 1 mg epinephrine, 1 mg atropine, and 1 L of colloid intravenous fluid bolus before return of spontaneous circulation. The patient was intubated and transferred to the intensive care unit. In the intensive care unit, the patient’s extremities were cool to touch with nonpalpable peripheral pulses. Femoral arterial line and RIJV CVC were inserted. Central venous pressure was 25 mm Hg, and arterial blood pressure was 85/46, which led to initiation of norepinephrine infusion after intravenous fluids were administered.

Repeat serum investigations revealed Hb 6.6 g/dL, platelets 49 × 10^9^/L, PT 40 (INR 4.2), APTT >123 (APTT ratio >4.2), and fibrinogen of 0.3 g/L. Deranged liver function test with ALT 852 U/L, aspartate aminotransaminase 717 IU/L, and albumin 13 g/L. Creatinine 119 µmol/L, urea 4.4 mmol/L, and potassium 4.3 mmol/L. ABG showed severe metabolic lactic acidosis, pH 6.68, PaO_2_ 24 kPa, PaCO_2_ 5.49 kPa, HCO_3_ 6.9 mmol/L, BE negative 27, and a lactate of 12.4 mmol/L. Within 4 hours of arrival in the emergency department, the patient had received 1 liter of crystalloids, 2.5 liters of colloids, 6 units of packed red blood cells, 4 units of Octoplast, 1 pack of platelets, and 3 g of fibrinogen. After surgical evaluation in the intensive care unit, the patient was emergently taken for an exploratory laparotomy given the drop in hemoglobin and distended abdomen on exam.

**Figure 1.**
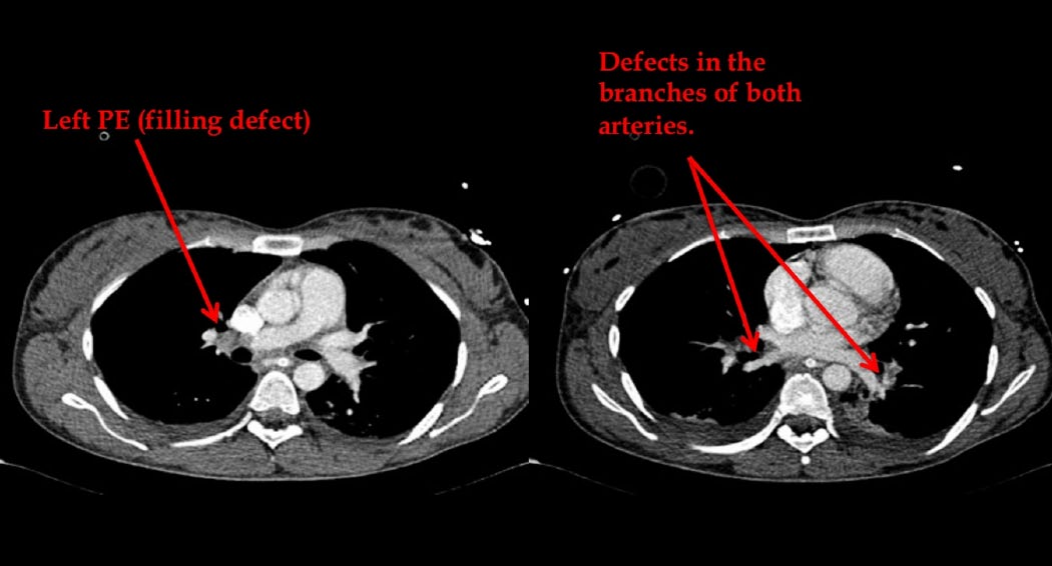
Slides from CT chest that show the left PE and defects in the branches of both pulmonary arteries.

**Figure 2.**
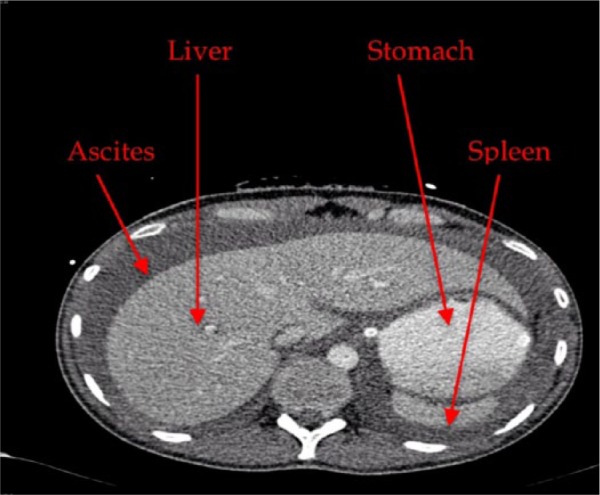
A slide from CT abdomen that shows that the patient is very thin with large ascites and very large liver.

The D-dimer returned while the patient was in the operating room and was 21.11 µg/mL (normal range <0.4). Findings at laparotomy were as follows: large amount of ascetic fluid in peritoneal cavity, firm congested and very enlarged liver, normal small and large bowel, no blood in peritoneal cavity, and no abnormality in pelvis. In view of the very enlarged firm and congested liver, it was decided to transfer the patient to the liver unit in a tertiary center. On arrival to the liver unit in the tertiary center a transthoracic echo (TTE) was done, which showed diastolic flattening of the ventricular septum in keeping with right ventricular overload. There was dilatation of the right ventricle and evidence of severe pulmonary hypertension. The RVSP was measured to be 77 mm Hg. There was a tricuspid regurgitation and the EF was 70%. There was no mention of McConnell’s sign in the report of TTE. The sudden elevation of the right ventricular pressure caused the congestion of the liver and the severe ascites. Computed tomography (CT) chest and CT liver portal phase (Figures 1 and 2) showed large thrombus in the right pulmonary artery and a thrombus in the two third order branches of the left lower lobe pulmonary artery. The right heart chambers were dilated. There was a gross ascites. The liver parenchyma appeared normal; however, the hepatic veins were mildly engorged. The portal vein was patent with no evidence of pelvic or inferior vena cava venous thrombosis.

Following discussion with cardiologist and interventional radiologist, it was decided not to proceed with embolectomy, and heparin infusion was commenced, which was converted to therapeutic low-molecular-weight heparin the following day. The patient’s condition improved, inotropes were weaned, and the patient was extubated within 1 day of arrival to the tertiary hospital. The patient was transferred back to the district hospital after spending 2 days in the tertiary center.

The precipitating cause in this case was the contraceptive pill. The patient was discharged home on warfarin.

## Discussion

Venous thrombosis is always a severe disease and is often fatal, because fragments of the thrombi may detach and occlude branches of the pulmonary artery. . . . The occlusion of the main branches of the pulmonary artery causes a striking rise of the blood pressure in these vessels. This rise—which the right heart must fight in order to ensure circulation—may sometimes lead to cardiac arrest.

—Picot 1884, *Lecous de Clinique Médicale*^1^

Massive PE is a life-threatening condition. In the International Cooperative Pulmonary Embolism Registry (ICOPER) of 2454 consecutive patients from 7 countries, 4.2% had massive PE.^2^ The Task Force Report clinically define massive PE as consisting of shock and/or hypotension (defined as a systolic blood pressure <90 mm Hg or a pressure drop of ≥40 mm Hg for >15 minutes if not caused by new-onset arrhythmia, hypovolemia, or sepsis). Anatomically massive PE is defined as a >50% obstruction of the pulmonary vasculature or the occlusion of 2 or more lobar arteries. From reported series, it is evident that the combination of embolus size and the cardiopulmonary function necessary to produce shock is associated with a mortality rate of approximately 30%. Massive PE that produces cardiac arrest has a mortality rate of at least 70% in reported series.^1^

The typical presentation of PE is with dyspnea (80%), chest pain (52%), cough (20%), hemoptysis (11%), and syncope (19%),^3^ while the atypical presentation is abdominal pain,^4-6^ high fever,^7^ new onset atrial fibrillation, and disseminated intravascular coagulation.^8^ When PE is present with abdominal pain, it is normally associated with other typical presentations of PE.

A 1957 review of 90 patients with PE described 6 (6.7%) of these patients presenting with abdominal pain as the primary complaint. Only 2 of these had “an acute surgical abdomen”; one patient in this series continued on to laparotomy.^9^ Smith^10^ described a case report of a 28-year-old woman who presented with an acute surgical abdomen that required abdominal exploration but the patient died in the recovery room. At autopsy it was revealed that the patient had multiple bilateral pulmonary emboli in large and medium-sized arteries.^10^ The mechanism for the abdominal pain has been suggested to be due to diaphragmatic pleurisy resulting from pulmonary infarction in the lung bases.^11^ Israel and Goldstein^9^ proposed that the pain may be due to passive hepatic congestion secondary to acute right ventricular dysfunction. Similarly, Kounis^12^ postulated distension of Glisson’s capsule and bowel edema as mechanisms of abdominal pain resulting from massive congestion. In a review of the pathogenesis of PE, Gorham^11^ also felt that pain might be due to tension on sensory nerve endings in the parietal pleura and hypoalgesia of intercostal muscles. In our patient the presentation of surgical acute abdomen is atypical of PE, but despite the severe cardiovascular collapse the patient had very high central venous pressure due to right ventricular failure. The TTE appearance of large PE includes right ventricular dilatation and hypokinesis, abnormal motion of the interventricular septum, tricuspid regurgitation, and lack of collapse of the inferior vena cava during inspiration.^13^ A bedside TTE would have revealed all of the above features of massive PE. Therefore, an elevated central venous pressure, presence of hepatomegaly, and an elevated D-dimer in a previously healthy young patient may have been clues to the diagnosis of massive PE.

### Why Should an Emergency Physician Be Aware of This?

This case highlights the fact that acute surgical abdomen and distension could be the only presenting complaint in massive pulmonary embolism. Emergency department physicians should be aware of such extreme presentation of pulmonary embolism.

## References

[1] Wood KE Major pulmonary embolism. Chest. 2002;121:877-905. 11888976 10.1378/chest.121.3.877

[2] Goldhaber SZ Visani L De Rosa M Acute pulmonary embolism: clinical outcomes in International Cooperative Pulmonary Embolism Registry (ICOPER). Lancet. 1999;353:1386-1389. 10227218 10.1016/s0140-6736(98)07534-5

[3] Torbicki A Perrier A Konstantinides S Guidelines on the diagnosis and management of acute pulmonary embolism. Eur Heart J. 2008;29:2276-2315. 18757870 10.1093/eurheartj/ehn310

[4] Unluer EE Denizbasi A Pulmonary embolism case presenting with upper abdominal and flank pain. Eur J Emerg Med. 2003;10:135-138. 12789072 10.1097/01.mej.0000072621.17469.c4

[5] vonPohle WR Pulmonary embolism presenting as acute abdominal pain. Respiration. 1996;63:318-320. 8885008 10.1159/000196569

[6] Kaminski N Lossos IS Ben-Sira L Laxer U Jaffe R Flank pain as a presentation of pulmonary embolism. Respir Med. 1995;89:65-66. 7708985 10.1016/0954-6111(95)90075-6

[7] Murray H Ellis G Blumenthal D Sos T Fever and pulmonary thromboembolism. Am J Med. 1979;67:232-235. 463928 10.1016/0002-9343(79)90396-6

[8] Stahl R Javid J Lackner H Unrecognized pulmonary embolism presenting as disseminated intravascular coagulation. Am J Med. 1984;76:772-778. 6720724 10.1016/0002-9343(84)90985-9

[9] Israel HL Goldstein F The varied clinical manifestations of pulmonary embolism. Ann Intern Med. 1957;47:202-226. 13459102 10.7326/0003-4819-47-2-202

[10] Smith DC Pulmonary embolism presenting as an acute surgical abdomen. J Emerg Med. 1996;14:715-717. 8969992 10.1016/s0736-4679(96)00182-5

[11] Gorham L A study of pulmonary embolism. Part III. The mechanism of pain, based on a clinico-pathological investigation of 100 cases of minor and 100 cases of massive embolism of the pulmonary artery. Arch Intern Med. 1961;108:140-148. 10.1001/archinte.1961.03620090090011 13707243

[12] Kounis N Pulmonary embolism with neurological and abdominal manifestations. Practitioner. 1979;223:115-116. 482240

[13] Goldhaber SZ Echocardiography in management of pulmonary embolism. Ann Intern Med. 2002;136:691-700. 11992305 10.7326/0003-4819-136-9-200205070-00012

